# Water disinfection by-products and the risk of specific birth defects: a population-based cross-sectional study in Taiwan

**DOI:** 10.1186/1476-069X-7-23

**Published:** 2008-06-02

**Authors:** Bing-Fang Hwang, Jouni JK Jaakkola, How-Ran Guo

**Affiliations:** 1 Department and Graduate Institute of Occupational Safety and Health, College of Public Health, China Medical University, No.91 Hsueh-Shih Road, Taichung 40402, Taiwan, ROC; 2 Department of Occupational and Environmental Medicine, National Chung Kung University, No. 138 Sheng-Li Road, Tainan 70428, Taiwan, ROC; 3 Department of Health Care Administration, Diwan College of Management, No. 87-1 Nan-Shih Li, Tainan 72153, Taiwan, ROC; 4 The Institute of Occupational and Environmental Medicine, The University of Birmingham, Edgbaston, Birmingham B15 2TT, UK

## Abstract

**Background:**

Recent findings suggest that exposure to disinfection by-products may increase the risk of birth defects. Previous studies have focused mainly on birth defects in general or groups of defects. The objective of the present study was to assess the effect of water disinfection by-products on the risk of most common specific birth defects.

**Methods:**

We conducted a population-based cross-sectional study of 396,049 Taiwanese births in 2001–2003 using information from the Birth Registry and Waterworks Registry. We compared the risk of eleven most common specific defects in four disinfection by-product exposure categories based on the levels of total trihalomethanes (TTHMs) representing high (TTHMs 20+ μg/L), medium (TTHMs 10–19 μg/L), low exposure (TTHMs 5–9 μg/L), and 0–4 μg/L as the reference category. In addition, we conducted a meta-analysis of the results from the present and previous studies focusing on the same birth defects.

**Results:**

In multivariate logistic regression analysis the risk of ventricular septal defects (adjusted odds ratio 1.81, 95% confidence interval: 0.98 3.35), cleft palate (1.56. 95% CI: 1.00, 2.41), and anencephalus (1.96, 95% CI: 0.94, 4.07) were elevated in the high exposure compared to the reference category. In the meta-analysis, the summary odds ratio for ventricular septal defects (1.59, 95% CI: 1.21, 2.07) was consistently elevated.

**Conclusion:**

The present study suggests that prenatal exposure to disinfection by-products increases the risk of ventricular septal defects, cleft palate, and anencephalus. The evidence on ventricular septal defects is consistent in the three available studies.

## Background

Water chlorination is a widely used and efficient method to reduce the occurrence of water-borne diseases, and has been one of the most successful public health measures introduced in the 20^th ^century. In early 1970s, some volatile halogenated organic compounds, such as chloroform, were identified in chlorinated surface waters containing high levels of natural organic material [1]. Later many other disinfection by-products, such as other trihalomethanes (THMs), haloacetic acids, chlorophenols, chloral hydrate, and haloacetonitriles have been identified, most from the process of chlorination, but also from chloramination, chlorine dioxide disinfection, and ozonation [2]. Generally, the THMs, including chloroform, bromodicholormethane, dibromochlormethane, and bromoform are the most prevalent in chlorinated surface water [3]. They are routinely measured throughout water works in Taiwan.

A recent meta-analysis of the 5 studies published by the end of 2001 indicated that exposure to chlorination by-products may increase the risk of birth defects in general, especially neural tube and urinary tract defects [4-9]. More recently a Swedish study provided evidence of an elevated risk of cardiac defects [10], whereas two Californian case-control studies of neural tube defects, cleft lip, and cleft palate provided inconsistent results [11]. In a Norwegian nationwide cross-sectional study, the risk of ventricular septal defects, cleft lip, and obstructive urinary tract defects were related to exposure to disinfection by products [12]. A recent study in England and Wales reported that the risk of ventricular septal defects was associated with exposure to disinfection by products [13].

We conducted a cross-sectional study of all Taiwanese births in years 2001–2003 to assess the effect of water disinfection by-products on the most common specific birth defects. We also synthesized quantitatively our results with the five previous epidemiologic studies [11-14], which focused on specific birth defects.

## Methods

### Study population

The source population comprised of all 721,289 newborns registered by the Taiwanese Birth Registry from 2001 to 2003. We excluded 325,240 newborns due to insufficient information on disinfection practice in the municipality where the mother was living during pregnancy. We focused on five water regions, which were served by only one type of water treatment plant. The final study population included 396,049 infants. The study protocol was approved by the Institutional Review Board of Diwan College of Management, and it complied with the principles outlined in the Helsinki Declaration [15].

### Health outcomes

We assessed the effect of disinfection by-products on the risk of the eleven most common specific birth defects according to the International Classification of Diseases, ninth revision (ICD-9), including anencephalus (740.0), hydrocephalus (741.0), ventricular septal defects (745.4), atrial septal defects (745.5), Tetralogy of Fallot (745.2), cleft palate (749.0), cleft lip (749.1), renal agenesis & dysgenesis (753.0), obstructive urinary tract defects (753.2), hypospadias (752.61), and chromosome anomalies (758).

According to the law the parents are required to report all births to the Taiwan Local Household Registry, which is managed by both the Department of Interior Affairs and the Department of Health. In addition, Taiwanese pregnant women are almost all covered by national health insurance (>99%) and access to prenatal care is free and good (at least 10 visits during pregnancy). Birth defects were mostly diagnosed by physician, most often by paediatrician using ultrasound. As a result, birth defects that were diagnosed after 7 days of age and gestational age less than 20 weeks, and induced abortions due to birth defects are not included in the registry. The validation of the Taiwanese birth registration showed a low percentage of missing information (1.6%) and high levels of validity (sensitivity and specificity was 92.8%, and 99.6% respectively) and reliability (Cohen's k statistics was 0.92) [16].

### Exposure assessment

Assessment of exposure was based on municipal-level water quality information on concentrations of total trihalomethanes (TTHMs), and the mother's place of residence during pregnancy. One or more waterworks serve each municipality and the water treatment plants seldom serve across the municipality borders. The Taiwanese water supply system is quite simple. Two hundred water treatment plants from Taiwanese Water Supply Corporation (TWSC) serving about 21 million people (90%) chlorinate their water, and privately owned wells (groundwater) serving about 2 million people (10%) do not use chlorination.

The general hypothesis was that exposure to disinfection by-products through tap water increases the risk of birth defects. We divided water treatment plants according to the levels of total trihalomethanes (TTHMs) (in μg/L) as a quantitative measure of the water disinfection by-products. The TTHM level is recorded routinely in most of the water treatment plants. Under the regulations operating during the study period, the standard sampling frequency for TTHMs was a minimum of four samples per year for each water treatment plant. We assessed exposure by calculating a weighted average of the modeled quarterly TTHM estimates for the appropriate water treatment plants during the date of conception and the date of birth. The weighting was based on the proportion of the trimester falling into each quarterly period. The number of measurements, and distribution of TTHMs between three exposure categories and reference category are shown in Table 1. We focused on five water regions where the process of disinfection was chlorination, rather than chloramination, chlorine dioxide disinfection or ozonation. In general, these regions reported similar overall prevalence of any birth defect (Table 2).

**Table 1:** The number of measurements and distribution of TTHMs between three exposure categories and reference category.

Exposure categories	No of samples	Mean ± SD (μg/L)	Minimum (μg/L)	25 percentile (μg/L)	Median (μg/L)	75 percentile (μg/L)	Maximum (μg/L)
Reference (TTHMs (0–4 μg/L)	528	3.64 ± 0.95	.85	3.50	3.80	4.06	4.96
Low (TTHMs 5–9 μg/L)	168	5.57 ± 0.96	5.00	6.25	7.15	8.05	9.50
Medium (TTHMs 10–19 μg/L)	240	16.48 ± 2.94	10.68	14.95	15.57	17.57	19.53
High (TTHMs 20 ± μg/L)	228	23.24 ± 2.27	20.35	21.90	22.70	24.90	32.65

**Table 2:** The prevalence of any birth defect by water region.

Water region	No. of birth	No. with any birth defects	Prevalence (%)
Region I	111372	717	0.6
Region II	106435	513	0.5
Region III	49503	255	0.5
Region IV	53211	293	0.6
Region V	75528	378	0.5

### Covariates

We used routine birth registry data to construct the following covariates: gender of infant (male; female), maternal age (<20 years; 20–34 years; >= 35 years), plurality (singleton; and multiple birth), and maternal health status defined as the presence of any of the following diseases or conditions: diabetes mellitus, anemia (HCT< 0.30/HGB<0.10), cardiac disease, acute or chronic lung disease, genital herpes, hydraminios/oligohydramnios, chronic hypertension, pregnancy-associated hypertension, eclampsia, imcompetent cervix, renal disease, Rh sensitization, uterine bleeding (yes; no). We received municipal level data from the Department of Household Registration Affairs, Taiwanese Population Data services, which were used to construct municipal level population density, which is a measure of the proportion of urban population in the municipality.

### Statistical methods

We estimated the prevalence (%) of the birth defects with 95% confidence intervals based on binomial distribution. We compared the risk of birth defects in three exposure categories (TTHMs 20+ μg/L; TTHMs 10–19 μg/L; TTHMs 5–9 μg/L) to the risk in the reference category with the lowest concentrations of TTHMs (0–4 μg/L). We used prevalence odds ratio as a measure of association and we applied logistic regression to estimate the adjusted odds ratios. The goodness of fit was tested with likelihood ratio tests (LR) to assess whether or not a variable contributes significantly to the model. First, we fitted a full model with a complete set of covariates. To elaborate sources of confounding, we fitted models with different combinations of covariates and compared the effect estimates from models with and without the covariate of interest. If the adjusted results differed from unadjusted results by > 10%, the variable was included in the model. To evaluate the effect modification, we systematically compared effect estimates on different levels of covariates. We tested the trend in the exposure-outcome relations in logistic regression by fitting an ordinal scale exposure variable (0,1,2,3) based on the exposure categories.

We searched the Medline data base from 1966 through Novmember 2007, using the following key words: (water chlorination OR trihalomethanes OR disinfection by-products) AND (birth defects). In addition, we searched primary references from the identified publications and reviewed manually issues of the Archives of Environmental Health, Environmental Health Perspectives, Environmental Research, and Epidemiology. We considered all epidemiological studies that assessed the relation between exposures to chlorination disinfection by-products, either directly or indirectly, and specific birth defects.

Three authors independently reviewed the articles, extracted data, and assessed the validity of the studies. We applied the following inclusion criteria on the basis of the type of study, study population, exposure definition, and outcome definition. We accepted *a priori *all studies with individual as the unit of observation, including cross-sectional, cohort and case-control studies.

Finally, we conducted a meta-analysis of the present and the other available studies of specific birth defects in relation to water chlorination [11-14]. We calculated summary odds ratios using both the fixed-effects and random-effects models. The fixed-effects model was calculated using the Mantel-Haenszel method with inverse variances of individual effect estimates as weights [17]. The random-effects models were calculated using the method of DerSimonian and Laird [18]. We calculated summary odds ratios using the estimates from the contrast between the highest and the reference category. We studied heterogeneity of the study-specific effect estimates by plotting the measures of effect and applying Q statistic. We elaborated the heterogeneity between the specific effect estimates, but presented systematically summary estimates from both fixed and random-effects models to offer readers a possibility for their own informed judgment. We also performed sensitivity analysis with and without the study with the largest sample size to identify the impact of this individual study on the results.

## Results

Among 369,049 newborns in the study population, we identified 2,148 births (0.6%) with one or several birth defects of interest. Table 3 displays the study population according to the exposure categories. The municipalities in the high exposure category had a lower population density compared with the reference municipalities. Table 4 shows a comparison of the populations included and excluded from the analyses, and the total population. There were no statistically significant differences (χ^2 ^test; p > 0.05) between the characteristics of the included population and the total population.

**Table 3:** Characteristics of the study population (N = 396,049) according to the categories of exposure, Taiwan, 2001–2003.

Characteristic	TTHMs (0–4 μg/L) **Reference **N (%)	TTHMs (5–9 μg/L) **Low **N (%)	TTHMs (10–19 μg/L) **Medium **N (%)	TTHMs (20+ μg/L) **High **N (%)	Total N (%)
Total	181,985 (100%)	55,950 (100%)	82,797 (100%)	75,317 (100%)	396,049 (100%)
Gender of infant					
Male	95,027 (52.2%)	29,128 (52.1%)	43,155 (52.1%)	39,413 (52.3%)	206,723(52.2%)
Female	86,958 (47.8%)	26,822 (47.9%)	39,642 (47.9%)	35,904 (47.7%)	189,326 (47.8%)
Maternal age					
<20 years	6,419 (3.5%)	1,838 (3.3%)	3,499 (4.2%)	3,461 (4.6%)	15,217 (3.8%)
20–34	156,087 (85.8%)	46,871 (83.8%)	72,402 (87.4%)	65,947 (87.6%)	341,307 (86.2%)
35-	19,476 (10.7%)	7,241 (12.9%)	6,893 (8.3%)	5,909 (7.8%)	39,519 (10.0%)
Maternal diabetes mellitus					
Yes	418 (0.2%)	189 (0.3%)	179 (0.2%)	144 (0.2%)	930 (0.2%)
No	181,567 (99.8%)	55,761 (99.7%)	82,618 (99.8%)	75,173 (99.8%)	395,119 (99.8%)
Plurality					
Singleton	176,791 (97.1%)	54,383 (97.2%)	80,755 (97.5%)	73,442 (97.5%)	385,371 (97.3%)
Multiple birth	5,194 (2.9%)	1,567 (2.8 %)	2,042 (2.5%)	1,875 (2.5%)	10,678 (2.7%)
Population density (no of people/km^2^)*					
<1000	29,881 (16.4%)	6,697 (12.0%)	20,387 (24.6%)	26,392 (35.2%)	83,357 (21.1%)
1000–5000	71,005 (39.0%)	27,257 (48.7%)	23,569 (28.5%)	31,823 (42.5%)	153,654 (38.8%)
>5000	81,099 (44.6%)	21,996 (39.3%)	38,841 (46.9%)	16,738 (22.3%)	158,674 (40.1%)

**Table 4:** Characteristics of included population, excluded population, and total population in Taiwan 2001–2003.

Characteristics	Included population		Excluded population		Total population	
	N	%	N	%	N	%
Total	396,049	100	325,240	100	721,289	100
Gender of infant						
Male	206,723	52.2	170,385	52.4	377,108	52.3
Female	189,326	47.8	154,524	47.6	343,850	47.7
Maternal age						
<20 years	15,217	3.8	16,223	5.0	31,440	4.4
20–34	341,307	86.2	282,381	86.8	623,688	86.5
35-	39,519	10.0	26,631	8.2	66,150	9.2
Maternal diabetes mellitus						
Yes	930	0.2	876	0.3	1,806	0.3
No	395,119	99.8	324,364	99.7	719,483	99.7
Plurality						
Singleton	385,371	97.3	316,903	97.4	702,274	97.4
Multiple birth	10,678	2.7	8,337	2.6	19,015	2.6
Population density (no of people/km^2^)*						
<1000	83,357	21.1	94,304	29.5	177,661	24.8
1000–5000	153,654	38.8	144,069	45.0	297,723	41.6
>5000	158,674	40.1	81,817	25.6	240,491	33.6

Table 5 shows that the prevalence of any birth defect was not directly related to the level of exposure. Further, the risks of anencephalus (adjusted OR 1.96, 95% CI: 0.94, 4.07), and ventricular septal defects (1.81, 95% CI: 0.98, 3.35), and cleft palate (1.56, 95% CI: 1.00, 2.41) were substantially higher in the high exposure category compared with the reference category. The effect estimates for obstructive urinary tract defects (1.44, 95% CI: 0.66, 3.14) and renal agenesis and dysgenesis (1.27, 95% CI: 0.69, 2.33) were slightly elevated. The exposure-response pattern for anencephalus was inconsistent with an elevated effect estimate in low but not in medium exposure category. The risk of ventricular septal defects was elevated only in the high exposure category, whereas the risk of cleft palate showed an exposure-response pattern (logistic regression test for trend: p = 0.03). The effect estimate for chromosome anomalies was slightly elevated in low (1.25, 95% CI: 0.95, 1.65), but not in medium (0.93, 95% CI: 0.70, 1.24) and high exposure categories (0.90, 95% CI: 0.66, 1.24), as compared with the reference category. In contrast, a negative assocation was found for hypospadias.

**Table 5:** Prevalences and prevalence odds ratios of the most common birth defects according to exposure to trihalomethanes in Taiwan 2001–2003.

Outcomes		TTHM	TTHM	TTHM	TTHM
		0–4 μg/L	5–9 μg/L	10–19 μg/L	20+ μg/L
		Reference	Low	Medium	High
Any birth defect					
N	2,148	978	368	421	381
P (%)	0.540	0.540	0.660	0.510	0.510
OR		1.00	1.23	0.95	0.94
aOR (95% CI)		1.00	1.21 (1.07–1.36)	0.97 (0.86–1.08)	1.00 (0.89–1.13)
Anencephalus					
N	43	19	9	2	13
P (%)	0.011	0.010	0.016	0.002	0.017
OR		1.00	1.54	0.23	1.65
aOR (95% CI)		1.00	1.59 (0.72–3.52)	0.23 (0.05–1.01)	1.96 (0.94–4.07)
Hydrocephalus					
N	118	58	24	19	17
P (%)	0.030	0.032	0.043	0.023	0.023
OR		1.00	1.35	0.72	0.71
aOR (95% CI)		1.00	1.36 (0.85–2.20)	0.71 (0.42–1.20)	0.74 (0.43–1.28)
Ventricular septal defects					
N	59	27	6	8	18
P (%)	0.015	0.015	0.011	0.010	0.024
OR		1.00	0.72	0.65	1.61
aOR (95% CI)		1.00	0.74 (0.31–1.80)	0.65 (0.29–1.43)	1.81 (0.98–3.35)
Atrial septal defects					
N	19	8	5	2	4
P (%)	0.005	0.004	0.009	0.002	0.005
OR		1.00	2.03	0.55	1.21
aOR (95% CI)		1.00	2.15 (0.70–6.60)	0.53 (0.11–2.49)	1.33 (0.39–4.58)
Tetralogy of Fallot					
N	24	13	6	3	2
P (%)	0.006	0.007	0.011	0.004	0.003
OR		1.00	1.50	0.51	0.37
aOR (95% CI)		1.00	1.60 (0.61–4.23)	0.46 (0.13–1.61)	0.32 (0.07–1.47)
Cleft lip with/without palate					
N	358	155	55	84	64
P (%)	0.090	0.085	0.098	0.101	0.085
OR		1.00	1.15	1.19	1.00
aOR (95% CI)		1.00	1.15 (0.84–1.56)	1.20 (0.91–1.55)	0.98 (0.73–1.32)
Cleft palate					
N	129	52	15	28	34
P (%)	0.033	0.029	0.027	0.034	0.045
OR		1.00	0.94	1.18	1.58
aOR (95% CI)		1.00	0.94 (0.53–1.68)	1.17 (0.74–1.86)	1.56 (1.00–2.41)
Renal agenesis & dysgenesis					
N	76	33	14	13	16
P (%)	0.019	0.018	0.025	0.016	0.021
OR		1.00	1.38	0.87	1.17
aOR (95% CI)		1.00	1.33 (0.71–2.48)	0.92 (0.48–1.75)	1.27 (0.69–2.33)
Obstructive urinary tract defects					
N	49	19	10	10	10
P (%)	0.012	0.010	0.018	0.012	0.013
OR		1.00	1.71	1.16	1.27
aOR (95% CI)		1.00	1.65 (0.77–3.56)	1.24 (0.57–2.67)	1.44 (0.66–3.14)
Hypospadias					
N	72	43	8	14	7
P (%)	0.018	0.024	0.014	0.017	0.009
OR		1.00	0.61	0.72	0.39
aOR (95% CI)		1.00	0.59 (0.28–1.26)	0.76 (0.41–1.38)	0.47 (0.21–1.04)
Chromosome anomalies					
N	364	174	72	67	51
P (%)	0.092	0.096	0.129	0.081	0.068
OR		1.00	1.35	0.85	0.71
aOR (95% CI)		1.00	1.25 (0.95–1.65)	0.93 (0.70–1.24)	0.90 (0.66–1.24)
Down syndrome					
N	166	73	36	30	27
P (%)	0.042	0.040	0.064	0.036	0.036
OR		1.00	1.60	0.90	0.89
aOR (95% CI)		1.00	1.48 (0.99–2.21)	1.00 (0.65–1.54)	1.17 (0.74–1.83)
Trisomy 13					
N	14	4	2	6	2
P (%)	0.004	0.002	0.004	0.007	0.003
OR		1.00	1.63	3.30	1.21
aOR (95% CI)		1.00	1.56 (0.28–8.55)	3.36 (0.94–12.0)	1.12 (0.20–6.29)
Trisomy 18					
N	54	23	12	13	6
P (%)	0.014	0.013	0.021	0.016	0.008
OR		1.00	1.70	1.24	0.63
aOR (95% CI)		1.00	1.64 (0.81–3.30)	1.31 (0.66–2.59)	0.81 (0.33–2.02)

*cOR: crude odds ratio; aOR: adjusted odds ratio. Logistic regression analysis adjusting for maternal age, plurality, and population density.*Estimates not given for the following rare outcomes: spinal bifida (n = 14) microcephalus (n = 9); endocardial cushion defects (n = 7); transposition of great vessels (n = 12); choanal atresia (n = 2); dysplasia of lung (n = 9); congenital cystic kidney (n = 24).

Table 6 shows the characteristics of the present study and the five previous studies and gives the study-specific adjusted odds ratios for the available outcomes. In five of the studies chlorine was used in disinfection, whereas in the Swedish study chlorine dioxide was applied.

**Table 6:** Summary of the results from previous and present studies focusing on seven most common specific birth defect.

	Sweden 2000 [14]	Norway 2002 [12]	California 2003^(1) ^[11]	California 2003^(2) ^[11]	England and Wales 2007 [13]	Taiwan 2008
Type of study	Cross-sectional, population-based	Cross-sectional, population-based	Case-control	Case-control	Cross-sectional, population-based	Cross-sectional, population-based
Study Population	115,801 births	184,676 births	539 controls	481 conrols	2,605,226 births	396,409 births
No of birth Defects	1,842	5,764	538	265	22,828	2,148
Exposure	Chlorine dioxide	Chlorination, High color	TTHMs 50–74 μg/l	TTHMs 50–74 μg/l	TTHMs >= 60 μg/l	TTHMs >= 20 μg/l
Reference	None	No chlorination, Low color	TTHMs 0 μg/l	TTHMs 0 μg/l	TTHMs <30 μg/l	TTHMs 0–4 μg/l
Anencephalus		0.79 (0.15–4.23) (n = 46)	0.55 (0.26–1.10) (n = 164)	1.80 (0.76–4.40) (n = 89)		1.96 (0.94–4.07) (n = 43)
Hydorcephalus	1.50 (0.30–7.30) (n = 36)	2.70 (0.77–9.51) (n = 68)				0.74 (0.43–1.28) (n = 118)
Ventricular septal defects		1.81 (1.05–3.09) (n = 279)			1.43 (1.00–2.04) (n = 408)	1.81 (0.98–3.35) (n = 59)
Atrial septal Defects		0.92 (0.24–1.50) (n = 73)				1.33 (0.39–4.58) (n = 19)
Cleft lip with/without palate		0.85 (0.53–1.38) (n = 248)		1.90 (0.81–4.50) (n = 117)	0.94 (0.83–1.06) (n = 2,909)	0.98 (0.73–1.32) (n = 358)
Cleft palate		1.07 (0.35–3.27) (n = 95)		1.00 (0.32–3.40) (n = 58)	0.95 (0.76–1.10) (n = 1,929)	1.56 (1.00–2.41) (n = 129)
Obstructive urinary tract defects		1.99 (0.66–5.96) (n = 102)				1.44 (0.66–3.14) (n = 49)

* Sweden 2000 [14]; California 2003^(1)^: Shaw et. al. Study 1 [11]; California 2003^(1)^: Shaw et. al. Study 2 [11]; Norway 2007 [12]; England and Wales 2007 [13]; Taiwan 2008: the present study.

As shown in table 7, the summary odds ratios for ventricular septal defects (summary OR 1.25, 95% CI: 1.08, 1.46) provided consistent evidence of an increased risk, whereas the summary odds ratios for atrial septal defects, cleft lip with/without palate, and cleft palate provided consistent evidence of no effect. The sensitivity analysis for cleft palate revealed that the English-Welsh study was responsible for the heterogeneity. After exclusion of this study, the summary odds ratio was homogeneous and substantially elevated (fixed-effects model: 1.43 (95% CI: 0.97, 2.10, heterogeneity: p = 0.212).

**Table 7:** A comparison of the results from the present, Sweden, Norway, California, and England and Wales studies.

Birth defect	Study area	Fixed-Effects Model Summary OR (95 % CI)	Random-Effects Model Summary OR (95% CI)	Q-statistic/P-value*
Anencephalus	Norway 2002	1.13 (0.74–1.73)	1.15 (0.56–2.36)	7.55/0.056
	California 2003 ^(1)^			
	California 2003 ^(2)^			
	Taiwan 2008			
	Norway 2002	0.85 (0.51–1.43)	0.91 (0.40–2.51)	4.23/0.121
	California 2003 ^(1)^			
	California 2003 ^(2)^			
	Norway 2002	0.98 (0.61–1.59)	0.98 (0.38–2.51)	6.19/0.045
	California 2003 ^(1)^			
	Taiwan 2008			
	Norway 2002	1.73 (1.01–2.96)	1.73 (1.01–2.96)	0.96/0.619
	California 2003 ^(2)^			
	Taiwan 2008			
Cleft palate	Norway 2002	1.00 (0.87–1.15)	1.10 (0.82–1.48)	4.50/0.212
	California 2003 ^(2)^			
	Taiwan 2008			
	England and Wales 2007			
	Norway 2002	1.43 (0.97–2.10)	1.43 (0.97–2.10)	0.74/0.690
	California 2003 ^(2)^			
	Taiwan 2008			
	Norway 2002	0.95 (0.83–1.10)	0.95 (0.83–1.10)	0.05/0.976
	California 2003 ^(2)^			
	England and Wales 2007			
	California 2003 ^(2)^	1.00 (0.87–1.15)	1.13 (0.77–1.66)	4.49/0.106
	Taiwan 2008			
	England and Wales 2007			
Cleft lip with/without palate	Norway 2002	0.95 (0.85–1.06)	0.95 (0.85–1.06)	2.76/0.431
	California 2003 ^(2)^			
	Taiwan 2008			
	England and Wales 2007			
	Norway 2002	1.00 (0.78–1.27)	1.01 (0.75–1.37)	2.58/0.276
	California 2003 ^(2)^			
	Taiwan 2008			
	Norway 2002	0.95 (0.84–1.06)	0.97 (0.76–1.24)	2.71/0.258
	California 2003 ^(2)^			
	England and Wales 2007			
	California 2003 ^(2)^	0.96 (0.86–1.07)	0.98 (0.82–1.15)	2.54/0.281
	Taiwan 2008			
	England and Wales 2007			
Ventricular septal defects	Norway 2002	1.59 (1.21–2.07)	1.59 (1.21–2.07)	0.74/0.692
	Taiwan 2008			
	England and Wales 2007			
	Norway 2002	1.81 (1.21–2.71)	1.81 (1.21–2.71)	0.0001/0.99
	Taiwan 2008			
Hydrocephalus	Sweden 2000	0.95 (0.59–1.50)	1.21 (0.52–2.81)	3.76/0.152
	Norway 2002			
	Taiwan 2008			
Obstructive urinary tract defects	Norway 2002	1.61 (0.85–3.03)	1.61 (0.85–3.03)	0.22/0.638
	Taiwan 2008			
Atrial septal defects	Norway 2002	0.97 (0.61–1.52)	0.97 (0.61–1.52)	0.30/0.59
	Taiwan 2008			

*P < 0.05 indicates that a random-effects model is more appropriate.

*Sweden 2000 [14]; California 2003^(1)^: Shaw et. al. Study 1 [11]; California 2003^(1)^: Shaw et. al. Study 2 [11] Norway 2007 [12]; England and Wales 2007 [13]; Taiwan 2008: the present study.

The summary odds ratio for hydrocephalus, based on three studies, was slightly elevated (random effects model: summary OR 1.21, 95% CI: 0.52, 2.81, heterogeneity: p = 0.152) but showed some heterogeneity. The effect estimate for anencephalus (fixed-effects model: summary OR 1.13, 95% CI: 0.74, 1.73, heterogeneity: p = 0.056) was slightly elevated but the study-specific effect estimates were heterogeneous. The sensitivity analysis revealed that one of the Californian case-control studies was responsible for the heterogeneity. After exclusion of this study, the summary odds ratio was homogeneous and substantially elevated (fixed-effects model: 1.73 (95% CI: 1.01, 2.96, heterogeneity: p = 0.619).

## Discussion

Assessment of potential effects of exposure to disinfection by-products on the risk of specific birth defects is problematic because of rarity and diversity of the congenital malformations. Few previous studies [10-13] have focused on selected birth defects and only two have assessed the associations between exposure and the risk of all the most common specific birth defects [12,13].

Our results showed no consistent association between exposure and the risk of birth defects in general. Of the 11 specific birth defects studied, the risk of ventricular septal defects, cleft palate, and anencephalus were substantially elevated in the high exposure compared to the reference category. The meta-analysis of 7 birth defects together with the Norwegian, California and British data strengthened the evidence for ventricular septal defects.

### Validity of results

The present study had enough power to estimate the relations between exposure to disinfection by-products and the most common specific birth defects, which could be relevant adverse effects based on previous literature. The meta-analysis together with the Norwegian, Californian and British results improved the precision of the finding on ventral septal defects. The specific birth defects are expected to be also more homogenous as to their causal factors compared with birth defect groups. We excluded half of the births because of insufficient water quality data. This exclusion was unlikely to introduce selection bias, because it was made on municipality level and the characteristic of the excluded individuals did not differ substantially from the included, as shown in Table 4.

Our outcome assessment was based on birth registration, as in the vast majority of the previous studies of disinfection by-products and birth defects [5-8,10,12-14], rather than clinical examination for the purposes of the study. This is a source of misclassification, which is likely to be random, i.e. not related to the exposure of interest, and thus lead to underestimation of the effect estimates. The sources of misclassification could include variation in diagnostic criteria and errors in reporting information provided by physician or hospital. Important features in the Taiwan national health care system limit the amount of outcome misclassification. Taiwanese pregnant women are almost all covered by health insurance (>99%,) and access to prenatal care is free and good (at least 10 visits during pregnancy). The clinical surveillance of pregnancy begins at 1 month after conception and continues through 7 days after birth. Birth defects were mostly diagnosed by physician, most often by paediatrician using ultrasound. According to the law, all live births in Taiwan must be reported within 15 days after birth. In general, the birth defects might be underreported, because we did not include the birth defects that were diagnosed after 7 days of age, gestational age less than 20 weeks, and induced abortions due to birth defects. This underreporting was likely to be nondifferential, i.e. not related to exposure. In most situations non-differential misclassification of a binary outcome will produce bias toward the null, provided that the misclassification is independent of other errors [19].

A major challenge of this study was the imprecision of exposure assessment from using aggregate municipal measures for classifying individual exposures. We had no information on the amounts of beverage and tap water consumption by pregnant women and exposure to volatile disinfection by-products through inhalation and dermal absorption, which might introduce non-differential misclassification and decrease the accuracy of exposure assessment. Future exposure assessment should include exposure through multiple routes such as bathing, showering and swimming, as well as water consumption. Unfortunately, we do not have sufficient information on alcohol consumption, cigarette smoking, vitamin consumption, medication, and genetic factors. Adjustment for population density adjusted indirectly for municipal differences in these behavior factors, but also eliminated partly regional differences in TTHM levels between rural and urban. Residential mobility during pregnancy may be an issue in our study. Two studies conducted in the United States showed that 25% [20] and 37% [21] of women moved during pregnancy. We have no information on the change of residence during pregnancy between three exposure categories (TTHMs 20+ μg/L; TTHMs 10–19 μg/L; TTHMs 5–9 μg/L) and the reference category (TTHMs (0–4 μg/L) in Taiwan birth registration. Since the misclassification of exposure is likely to be non-differential related to the outcomes of interest, the level of such misclassification would likely bias the effect estimations toward to the null. We systematically carried out stratified analyses in different categories of exposure and other covariates to elaborate the residual confounding and potential effect modification. The stratified analyses did not indicate any major confounding or effect modification.

The interpretation of meta-analysis is more difficult when the specific effect estimates differ substantially from each other, especially if there are estimates both below and above unity. The random-effects model has become a standard approach to incorporate heterogeneity. We elaborated the heterogeneity between the specific effect estimates, but presented systematically summary estimates from both fixed and random-effects models to offer readers a possibility for their own informed judgment. We also made an attempt to explain the observed heterogeneity, although the small number of studies limited the applicability of this approach. The type of study, study populations, and outcome assessment were relatively similar between the summary studies, but there were different approaches to exposure assessment. The present and three previous studies using routine measurements of trihalomethanes [11,13] constituted a rather homogeneous group, but different exposure contrasting. Also the Swedish study used water source and chlorination practice as the basis for exposure assessment [14], and the Norwegian study was based on the amount of organic content expressed in color and the presence of chlorination, it was possible to use a similar contrast between the highest exposure category and the reference group of no exposure [12]. Additionally, the sensitivity analysis indicated that the largest studies had a substantial impact on the summary effect. [13].

### Synthesis with previous knowledge

Six [5-8,11,22] of the eight previous studies [5-8,10-12,22] have provided evidence of an increase in the risk of neural tube defects related to exposure to disinfection by-products. Two large case-control studies in California focusing on ancephalus and spina bifida did not provide a clear pattern of the relation of exposure to THMs [11]. A case-control study in New Jersey reported no significant association between exposure to THMs and the risk of spina bifida [22]. Interestingly in the present study the risk of anencephalus was elevated in the highest exposure category. The meta-analysis of the four available studies provided an inconclusive, heterogeneous summary estimate.

The previous findings on cardiac defects as a group have been heterogeneous and inconsistent [5,6,8,10,12,13]. In the present study, the risk of ventricular septal defects was almost two times higher in the high exposure category compared to the reference category (adjusted OR 1.81, 95% CI: 0.98, 3.35). This is consistent with the Norwegian study, which reported an exposure-related increase of ventricular septal defects (medium exposure: adjusted OR 1.63, 95% CI: 1.02, 2.58; high exposure 1.81, 1.05–3.09), and the British study (high exposure vs. reference: adjusted OR 1.43, 95% CI: 1.00, 2.04). The corresponding summary OR was 1.59 (95% CI: 1.21, 2.07). Recently the risk of ventricular septal defects has also been found to be related to the level of traffic-related air pollution [23].

Previous five studies [5,6,8,11-13] on cleft lip and palate defects have given heterogeneous and/or inconsistent results. In the present study, the risk of cleft palate was related to the levels of TTHMs with an exposure-response pattern, yielding adjusted OR of 1.17 (95% CI: 0.74, 1.86) for medium and 1.56 (95% CI: 1.00, 2.41) for highest exposure category. Similar results were reported also from the Norwegian and California studies [9,11], but the British study resulted in an adjusted OR of 0.95 (95% CI: 0.87, 1.10) [13]. The four studies gave the summary odds ratio of 1.00 (95% CI: 0.87, 1.15).

Previous studies conducted in Massachusetts [4] and Norway [8,12] have provided rather homogeneous and consistent evidence of an effect on urinary tract defects as a group. In the present and British [13] studies there were no association between exposure and urinary tract defects per se, the risks of a smaller group denoted as obstructive urinary tract defects (ICD-9: 753.2) and renal agenesis and dysgenesis (ICD-9: 753.0) were found to be slightly elevated both in the high and low exposure categories. The Norwegian study showed some evidence of an increased risk of urinary tract defects as a group and an exposure response pattern for the risk of obstructive urinary tract defects (ICD-8: 753.2) [12]. Urinary tract defects are rare and the effect estimate imprecise, and therefore the findings of the role of exposure to disinfection by-products remain inconclusive. A negative association was found for hypospadias. This could be explained by chance while unity was within the 95% confidence interval. Unfortunately the diagnosis of hypospadias may also be compromised because it is made within the 7 days from delivery. Therefor the results of hypospadias should be interpreted cautiously.

Our study and two recent studies conducted in Norway and in England and Wales suggest that prenatal exposure to disinfection by-products increases the risk of ventricular septal defects at much lower levels than found in United States [5,7], and Canadian [6] drinking water sources, probably explained by qualitative geographic differences in the levels of natural organic matter (disinfection by-products precursor) or higher concentration of other non-volatile disinfection by-products (eg. haloacetic acids). The present and the two previous studies from Norway and California suggest also an increased risk of cleft palate.

### Biologic mechanisms

The specific mechanisms for the effects of trihalomethanes (THMs) on the risk birth defects are still unknown. Some animal studies show reproductive and developmental toxicity of some of these compounds, such as chloroform (CF) and bromodichloromethanes (BDCM), when given at high doses [3]. There is evidence that metabolites of chloroform may accumulate in the amniotic fluid of pregnant mice [24]. In addition, BDCM can disrupt syncytiotrophoblast formation and inhibit chorionic gonadotrophin secretion in vitro [25]. This implies that the placenta is a likely target of BDCM toxicity in human and thus BDCM may have teratogenic effects on fetus.

An alternative explanation is that THMs may lead to birth defects via genetic damage to maternal gametes. For example, CF may be oxidatively metabolized and decomposed to electrophilic phosgene, which is more likely to react and bind to cell components including proteins, phospholipid polar heads, and reduced glutathione [26]. This may result in chromosomal abnormalities, enzymatic malfunction, and disruption of cellular membranes, all of which could interfere with uterine development or directly influence on the conceptus.

Although THMs are the most prevalent in chlorinated water, they are also markers of a complex mixture of disinfections by-products. Some animal studies also show reproductive and developmental toxicity of haloacetic acid, non-volotile disinfection by-products, such as dichloroacetic acid (DCAA) and trichloracetic acid (TCAA) [3]. Further detailed toxicological assessments of mixtures of chlorination by-products are also needed, as humans are most commonly exposed to complex mixtures of these compounds rather than to a single compound [27].

## Conclusion

The present study suggests that prenatal exposure to disinfection by-products increases the risk of ventricular septal defects, cleft palate, and anencephalus. The finding on ventricular septal defects is consistent with previous epidemiologic studies [12,13], which strengthens the weight of evidence. Our findings demonstrate the importance of focusing on specific birth defects, rather than using broad categories of outcomes based on organ systems.

## Abbreviations

CI: confidence interval; TTHMs: total trihalomethanes; ICD-9: International Classification of Disease, ninth revision; aOR: adjusted odds ratio

## Competing interests

The authors declare that they have no competing interests.

## Authors' contributions

BFH is responsible for study concept and design, integrity of the data, the accuracy of the data analysis, and drafting of the manuscript, JJKJ for critical revision of the manuscript for important intellectual content, HRG for study concept and design, and study supervision. All authors read and approved the final manuscript.
